# PCSK9 Promotes Atherosclerotic Plaque Instability by Inducing VSMC Ferroptosis through the YAP1–NUPR1 Axis

**DOI:** 10.34133/research.0922

**Published:** 2025-10-07

**Authors:** Yuting Cui, Yanyu Chen, HengJuan Li, Weizheng Zhang, Xin Wang, Mengdie Xia, Ni Gan, Yating Zhou, Man Li, Huayu Zhang, Qiong Xiang, Xi-Long Zheng, Gang Fan, Jing Yang, Juan Peng, Xiaoyan Dai, Zhihan Tang

**Affiliations:** ^1^ Institute of Cardiovascular Disease, Key Laboratory for Arteriosclerology of Hunan Province, Hunan Intern Scientific and Technological Cooperation Base of Arteriosclerotic Disease, School of Basic Medical Sciences, Hengyang Medical School, University of South China, Hengyang 421001, Hunan, China.; ^2^ Department of Pathophysiology, School of Medicine, Sun Yat-sen University, Shenzhen 518107, Guangdong, China.; ^3^ Department of Cardiovascular Medicine, the Second Affiliated Hospital, University of South China, Hengyang 421001, Hunan, China.; ^4^ The Second People’s Hospital of Hunan Province (Brain Hospital of Hunan Province), Changsha 410007, Hunan, China.; ^5^ Department of Metabolism and Endocrinology, the First Affiliated Hospital, Hengyang Medical School, University of South China, Hengyang 421001, Hunan, China.; ^6^ Hunan Provincial Key Laboratory of Regional Hereditary Birth Defect Prevention and Control, Changsha Hospital for Maternal & Child Health Care Affiliated to Hunan Normal University, Changsha 410000, Hunan, China.; ^7^ Departments of Biochemistry & Molecular Biology and Physiology & Pharmacology, Cumming School of Medicine, University of Calgary, Calgary, Alberta T2N 4Z6, Canada.; ^8^ Department of Urology, Shenzhen University Sixth Affiliated Hospital, Shenzhen Nanshan People’s Hospital, Shenzhen 518052, China.; ^9^ Department of Metabolism and Endocrinology, Shenzhen Nanshan People’s Hospital, The Sixth Affiliated Hospital of Shenzhen University Health Science Center, Shenzhen 518052, Guangdong, China.; ^10^ Clinical Research Institute, the Second Affiliated Hospital, University of South China, Hengyang 421001, Hunan, China.; ^11^ Department of Cardiology, Affiliated Nanhua Hospital, Hengyang Medical School, University of South China, Hengyang 421001, Hunan, China.

## Abstract

Atherosclerosis persists as a principal driver of global cardiovascular mortality and morbidity, and its sustained prevalence surge fuels the incidence of major adverse cardiovascular events (MACE). Plaque instability is a critical determinant of MACE, as fissure formation or rupture of vulnerable plaques can precipitate thromboembolic complications. In this study, we investigate a noncanonical role of proprotein convertase subtilisin/kexin type 9 (PCSK9) beyond its lipid regulatory function, focusing on its impact on vascular smooth muscle cells (VSMCs) in the context of plaque instability. Our results demonstrate that PCSK9 overactivity markedly promotes ferroptotic cell death in VSMCs, thereby exacerbating plaque vulnerability. Furthermore, we delineate the underlying mechanism: PCSK9 physically interacts with Yes-associated protein 1 and targets it for lysosomal degradation, which, in turn, suppresses the expression of nuclear protein 1. In conclusion, our findings unveil a novel role of PCSK9 in promoting plaque instability by driving ferroptosis in VSMCs, suggesting that targeting PCSK9 presents a potential avenue for plaque stabilization, thereby mitigating the incidence of major MACE.

## Introduction

Cardiovascular diseases (CVDs) remain among the leading causes of death and disability worldwide, and their prevalence continues to climb [1]. Unstable atherosclerotic plaques tend to develop fissures or rupture, events that account for the majority of acute coronary syndromes, myocardial infarctions, and strokes [2]. Vulnerable plaques are defined by their prototypical microarchitectural features: a thin fibrous cap, a substantial lipid-rich necrotic core, and dense infiltration of inflammatory cells [3–5]. Currently, beyond standard therapies for CVDs, there are no targeted interventions that directly strengthen plaque stability. Thus, unraveling the molecular drivers of plaque destabilization is crucial for developing novel strategies to combat plaque vulnerability. Recent advances in atherosclerosis research—including the use of innovative in vivo plaque models—are beginning to reveal new molecular targets for stabilizing plaques [6].

Proprotein convertase subtilisin/kexin type 9 (PCSK9) is a crucial regulator of cholesterol homeostasis, primarily known for orchestrating low-density lipoprotein receptor (LDLR) degradation via lysosomal trafficking, creating a sustained elevation of plasma LDL particles and accelerating atherosclerosis [7–9]. PCSK9 inhibitors have been introduced in clinical practice to lower lipid levels, and trials have demonstrated that these agents can significantly reduce ischemic cardiovascular events in high-risk patients [10,11]. Interestingly, beyond their lipid-lowering effects, PCSK9 inhibitors have been associated with increased vascular smooth muscle cell (VSMC) content and thicker fibrous caps in plaques—features indicative of improved plaque stability [12–14]. Furthermore, circulating PCSK9 levels positively correlate with necrotic core size in coronary lesions even after adjusting for low-density lipoprotein cholesterol (LDL-C) [15]. These findings imply that PCSK9 can directly influence plaque vulnerability through mechanisms independent of its canonical lipid-modulatory function. However, the specific molecular pathways by which PCSK9 promotes plaque destabilization remain poorly understood.

Cell death within atherosclerotic lesions is a prominent feature of advanced plaques and a key contributor to plaque destabilization [16–18]. In particular, the loss of VSMCs from the fibrous cap is known to weaken plaque structure and promote vulnerability [19,20]. Recent evidence indicates that ferroptosis—a regulated, iron-dependent form of cell death—in plaque macrophages can exacerbate atheroma instability [21], suggesting that ferroptosis may be an important driver of plaque vulnerability. Intriguingly, emerging studies have linked PCSK9 to the regulation of ferroptosis. For example, inhibiting PCSK9 was shown to modulate ferroptosis-related markers and attenuate myocardial ischemia/reperfusion injury [22], and bioinformatic analyses have found correlations between PCSK9 expression and ferroptosis-associated genes in abdominal aortic aneurysm lesions [23]. Collectively, these observations point to PCSK9 as a novel regulator of ferroptotic cell death with potential implications across CVDs. Given that PCSK9 is abundantly expressed in VSMCs within human atherosclerotic plaques [24,25], it is plausible that PCSK9 directly influences ferroptosis in VSMCs—although the precise mechanism linking PCSK9 to VSMC ferroptosis remains to be clarified.

In this study, we found a significant correlation between higher PCSK9 levels and markers of plaque instability in human atherosclerotic lesions. Through VSMC-specific PCSK9 overexpression in mice, we demonstrated that PCSK9 induces ferroptosis in VSMCs and thereby contributes to plaque destabilization. Mechanistically, PCSK9 interacts with Yes-associated protein 1 (YAP1), leading to YAP1’s degradation and subsequent repression of nuclear protein 1 (NUPR1) expression. Finally, inspired by the proteolysis-targeting chimera (PROTAC) strategy, we designed a small-molecule peptide called Cadd4, which effectively degraded PCSK9, reduced VSMC ferroptosis, and stabilized plaques in vivo. In summary, our findings indicate that targeting PCSK9 may represent a promising therapeutic strategy to stabilize atherosclerotic plaques and reduce the risk of cardiovascular events.

## Results

### Intraplaque PCSK9 protein levels are positively correlated with plaque vulnerability and are elevated in VSMCs of human vulnerable plaques

**Fig. 1.**
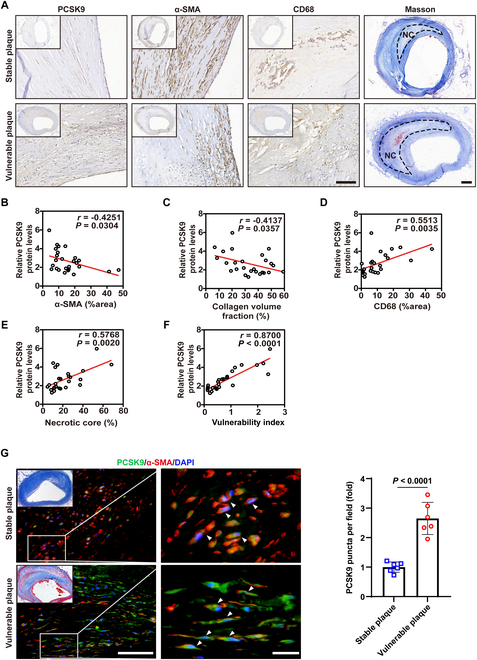
PCSK9 protein levels are associated with increased vulnerability of atherosclerotic plaques in humans. (A) Immunohistochemical staining for PCSK9, CD68, and α-SMA, as well as Masson staining, was performed in stable and vulnerable human plaques. The black continuous line marks the necrotic core area (NC). Scale bars from left to right: 100 and 500 μm. (B to F) Pearson correlations between PCSK9 expression and α-SMA (% area) (B), collagen volume fraction (%) (C), CD68 (% area) (D), necrotic core (% area) (E), and the vulnerability index (F) are presented. The vulnerability index is calculated as [CD68 (% area) + necrotic core (% area)]/[α-SMA (% area) + collagen volume fraction (%)] (*n* = 26 per group). (G) Immunofluorescent staining for PCSK9 (green) and α-SMA (red) in stable and vulnerable human plaques, with nuclei stained by DAPI (blue) (*n* = 6). The white arrowheads indicate colocalized cells. Scale bars from left to right: 75 and 20 μm. Statistical analysis was performed by unpaired Student’s *t* test (G).

To explore the relationship between PCSK9 expression and atherosclerotic plaque stability, we performed Masson’s trichrome staining and immunohistochemical analysis of α-SMA (smooth muscle marker), CD68 (macrophage marker), and PCSK9 in human atherosclerotic plaque specimens [26]. The results showed that PCSK9 expression was significantly higher in histologically vulnerable plaques (those with thin fibrous caps and large necrotic cores) compared to stable plaques (Fig. 1A). Vulnerable plaques were characterized by an increased CD68^+^ macrophage area, a larger necrotic core, a reduced α-SMA^+^ VSMC area, and a lower collagen content (Fig. 1A), consistent with features of plaque instability.

Pearson correlation analysis further revealed that intraplaque PCSK9 protein levels inversely correlated with both the α-SMA^+^ area (VSMC content) (Fig. 1B) and collagen fraction (Fig. 1C), but positively correlated with the CD68^+^ macrophage area (Fig. 1D) and the necrotic core area (Fig. 1E). Accordingly, PCSK9 levels showed a strong positive association with the calculated plaque vulnerability index—defined as [CD68 (% area) + necrotic core (% area)]/[α-SMA (% area) + collagen (% area)]—in these human plaques (Fig. 1F). Immunofluorescence co-staining confirmed a marked increase in PCSK9 expression specifically within VSMCs (α-SMA^+^ cells) of vulnerable plaques compared to stable plaques (Fig. 1G).

### VSMC-specific overexpression of PCSK9 exacerbates plaque vulnerability

To investigate the direct role of VSMC-derived PCSK9 in plaque instability, we generated transgenic mice with VSMC-specific PCSK9 overexpression (PCSK9^SMC OE^) using a Cre-loxP system (Fig. S1A). We confirmed successful PCSK9 overexpression in the aortas of PCSK9^SMC OE^ mice by RT-qPCR, Western blotting, and immunofluorescence, which showed elevated PCSK9 in the medial layer of the aorta relative to PCSK9^fl/fl^ control mice (Fig. S1B to D).

To induce atherosclerosis, we subjected mice to a single tail-vein injection of an AAV8 vector encoding a gain-of-function mutant mouse PCSK9 (D377Y) under the control of liver-specific promoter combined with 12 weeks of high-fat diet (HFD) feeding (Fig. S2A). Notably, plasma lipid profiles (triglycerides, total cholesterol, high-density lipoprotein cholesterol (HDL-C), and LDL-C) did not differ significantly between PCSK9^SMC OE^ mice and control mice during the experiment (Fig. S2B to E), indicating that any observed differences in plaque features are not due to systemic lipid level changes.

**Fig. 2.**
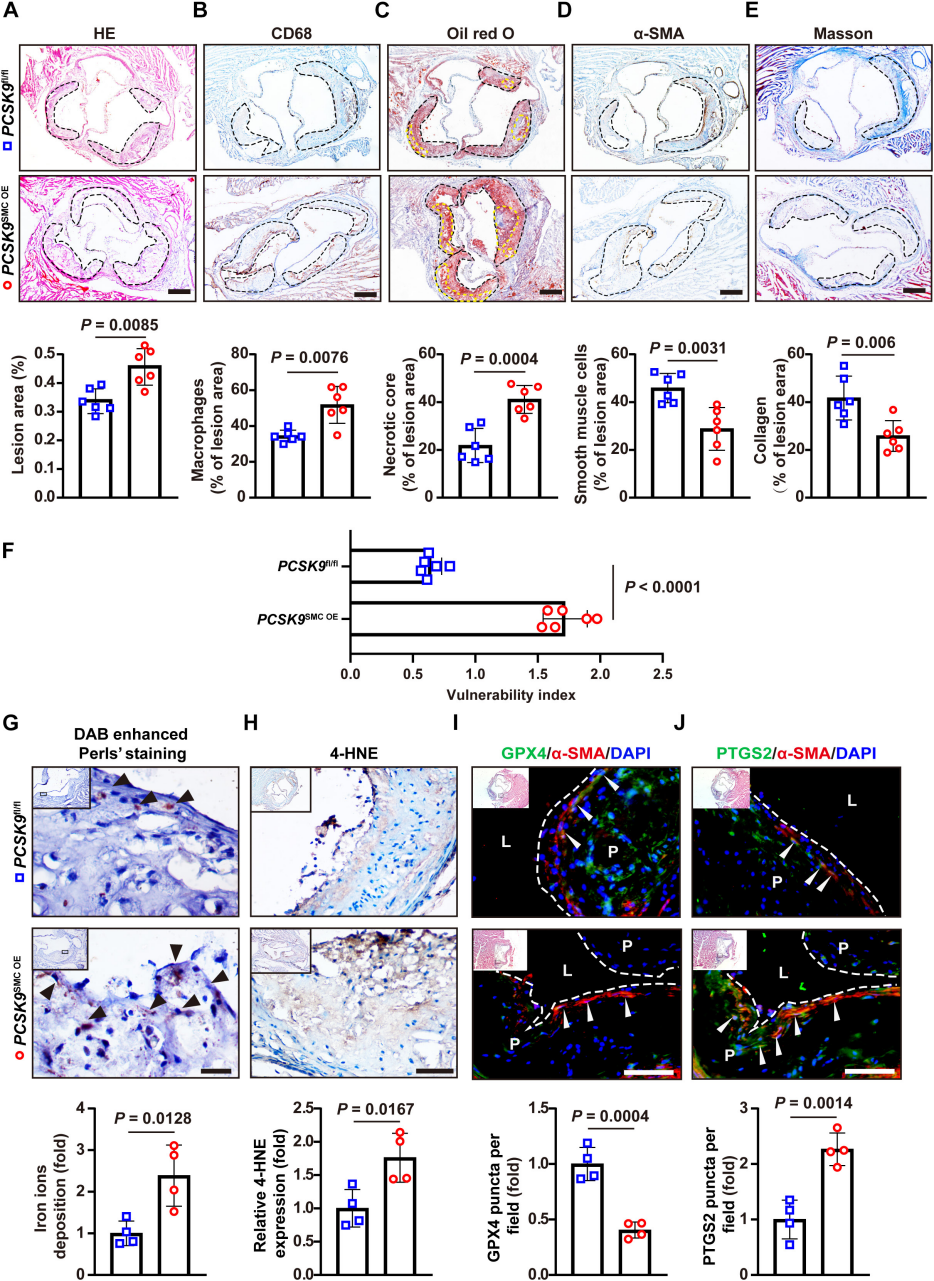
VSMC-specific overexpression of PCSK9 exacerbates plaque vulnerability and VSMC ferroptosis in vivo. (A) Hematoxylin and eosin (H&E) staining showed the plaque lesion area as a percentage of the vascular area (*n* = 6). Scale bar: 200 μm. (B) Immunohistochemical staining revealed the percentages of macrophages in the lesion area (*n* = 6). Scale bar: 200 μm. (C) Oil Red O staining (*n* = 6). Scale bar: 200 μm. (E) Masson staining (*n* = 6). Scale bar: 200 μm. (D) Immunohistochemical staining revealed the percentages of smooth muscle cells in the lesion area. (F) Vulnerability index of aortic root plaques (*n* = 6). The solid continuous line denotes the area of the atherosclerotic plaque. (G) Perls’ Prussian blue staining for iron, with black arrowheads indicating positive cells (*n* = 4). Scale bar: 20 μm. (H) Immunohistochemical staining of 4-HNE (*n* = 4). Scale bar: 50 μm. (I and J) Immunofluorescent staining for GPX4 and PTGS2 (green) was utilized to evaluate ferroptosis, with α-SMA (red) serving as a reference (*n* = 4). Nuclei were stained with DAPI (blue). White arrowheads indicate colocalized cells. L, lumen; P, plaque. Scale bar: 75 μm. Statistical analysis was performed by unpaired Student’s *t* test (A to J).

We next examined aortic root atherosclerotic plaques from these mice. Histological analyses (hematoxylin and eosin [H&E] staining for overall morphology, Oil Red O for lipids, Masson’s trichrome for collagen, and immunohistochemistry (IHC) for CD68 and α-SMA) demonstrated that PCSK9^SMC OE^ mice had significantly larger plaques than controls (Fig. 2A). Specifically, PCSK9 overexpression in VSMCs led to increased intraplaque CD68^+^ macrophage area (Fig. 2B) and a larger necrotic core (Fig. 2C), while reducing the α-SMA^+^ VSMC area (Fig. 2D) and collagen fraction (Fig. 2E) within plaques. Consequently, the plaque vulnerability index was markedly elevated in PCSK9^SMC OE^ mice (Fig. 2F). These findings demonstrate that VSMC-specific overexpression of PCSK9 exacerbates plaque instability in atherosclerosis, emphasizing a direct contribution of VSMC-derived PCSK9 to plaque vulnerability.

### PCSK9 overexpression induces VSMC ferroptosis in vivo

Next, we sought to determine how VSMC-derived PCSK9 accelerates plaque vulnerability. We performed transcriptomic analysis (RNA-seq) on primary VSMCs with PCSK9 overexpression and compared the differentially expressed genes (DEGs) to a published dataset associated with plaque instability (GSE120521). KEGG pathway enrichment analysis indicated that the ferroptosis pathway was significantly enriched among the DEGs (Fig. S3A and B). In line with this, aortic root plaques from PCSK9^SMC OE^ mice showed clear biochemical signs of ferroptosis: significantly higher levels of free iron (Fe^2+^) and 4-hydroxynonenal (4-HNE, a lipid peroxidation marker) compared to plaques from PCSK9^fl/fl^ mice (Fig. 2G and H). Likewise, VSMCs in PCSK9^SMC OE^ plaques had reduced expression of the anti-ferroptotic enzyme glutathione peroxidase 4 (GPX4) and increased expression of the pro-ferroptotic enzyme prostaglandin-endoperoxide synthase 2 (PTGS2, also known as COX-2) (Fig. 2I and J). These results demonstrate that PCSK9 overexpression triggers ferroptosis in VSMCs in vivo.

### PCSK9 promotes ferroptosis in VSMCs in vitro

**Fig. 3.**
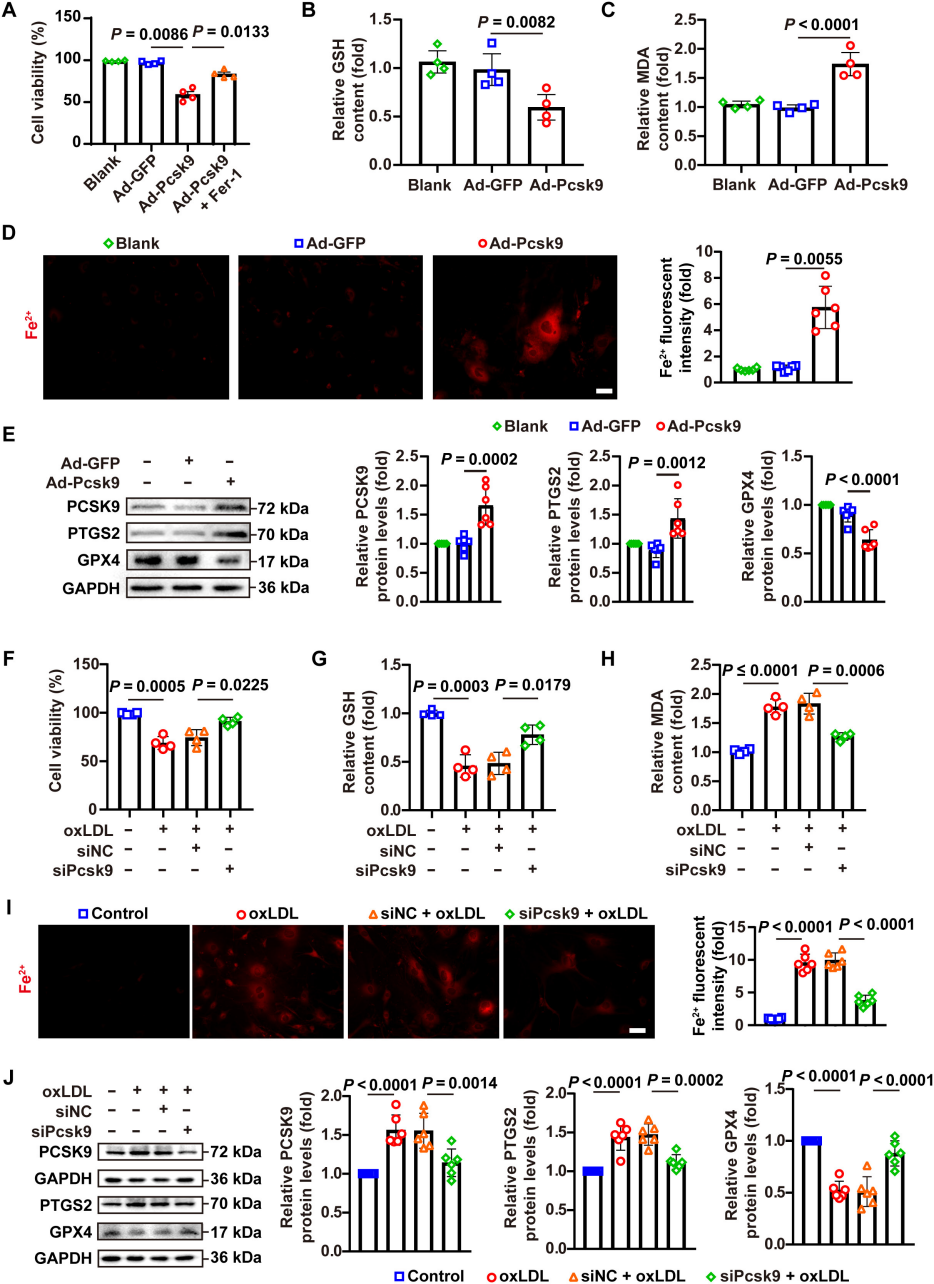
PCSK9 enhances ferroptosis in VSMCs in vitro. (A to C) Cell viability (A), glutathione (GSH) levels (B), and malondialdehyde (MDA) levels (C) were assessed in primary mouse VSMCs treated as indicated (*n* = 4). (D) Living cell FerroOrange staining in primary mouse VSMCs treated as indicated (*n* = 6). Scale bar: 50 μm. (E) Western blot analysis of the protein levels of PCSK9, PTGS2, and GPX4 in primary mouse VSMCs treated as indicated (*n* = 6). (F to H) Cell viability (F), GSH levels (G), and MDA levels (H) were measured in primary mouse VSMCs treated with siNC or siPcsk9 for 48 h and oxLDL (50 μg/ml) for 24 h (*n* = 4). (I) Living cell FerroOrange staining (*n* = 6). Scale bar: 50 μm. (J) Western blot analysis of the protein levels of PCSK9, PTGS2, and GPX4 in primary mouse VSMCs treated with siNC or siPcsk9 for 48 h and oxLDL (50 μg/ml) for 24 h (*n* = 6). Statistical analysis was performed by 1-way ANOVA (A to E) or 2-way ANOVA test (F to J).

To directly confirm the role of PCSK9 in inducing VSMC ferroptosis, we infected primary mouse VSMCs with an adenoviral vector encoding PCSK9 (Ad-PCSK9) or a control vector. Ferroptosis was evaluated by assessing cell viability and multiple ferroptotic markers, including GPX4 and PTGS2 protein levels, glutathione (GSH) content, malondialdehyde (MDA) levels, and ferrous iron (Fe^2+^) accumulation. A Cell Counting Kit-8 (CCK-8) cell viability assay showed that PCSK9 overexpression significantly reduced VSMC viability, and importantly, this cell death was rescued by the ferroptosis inhibitor ferrostatin-1 (Fer-1) (Fig. 3A), supporting that the cell death was ferroptotic in nature. Consistently, PCSK9-overexpressing VSMCs exhibited a sharp decrease in GSH levels alongside elevated intracellular MDA and Fe^2+^ (Fig. 3B to D). Western blot analysis further corroborated these findings: PCSK9 increased PTGS2 levels and decreased GPX4 levels in VSMCs (Fig. 3E).

### PCSK9 induces VSMC ferroptosis by down-regulating NUPR1

**Fig. 4.**
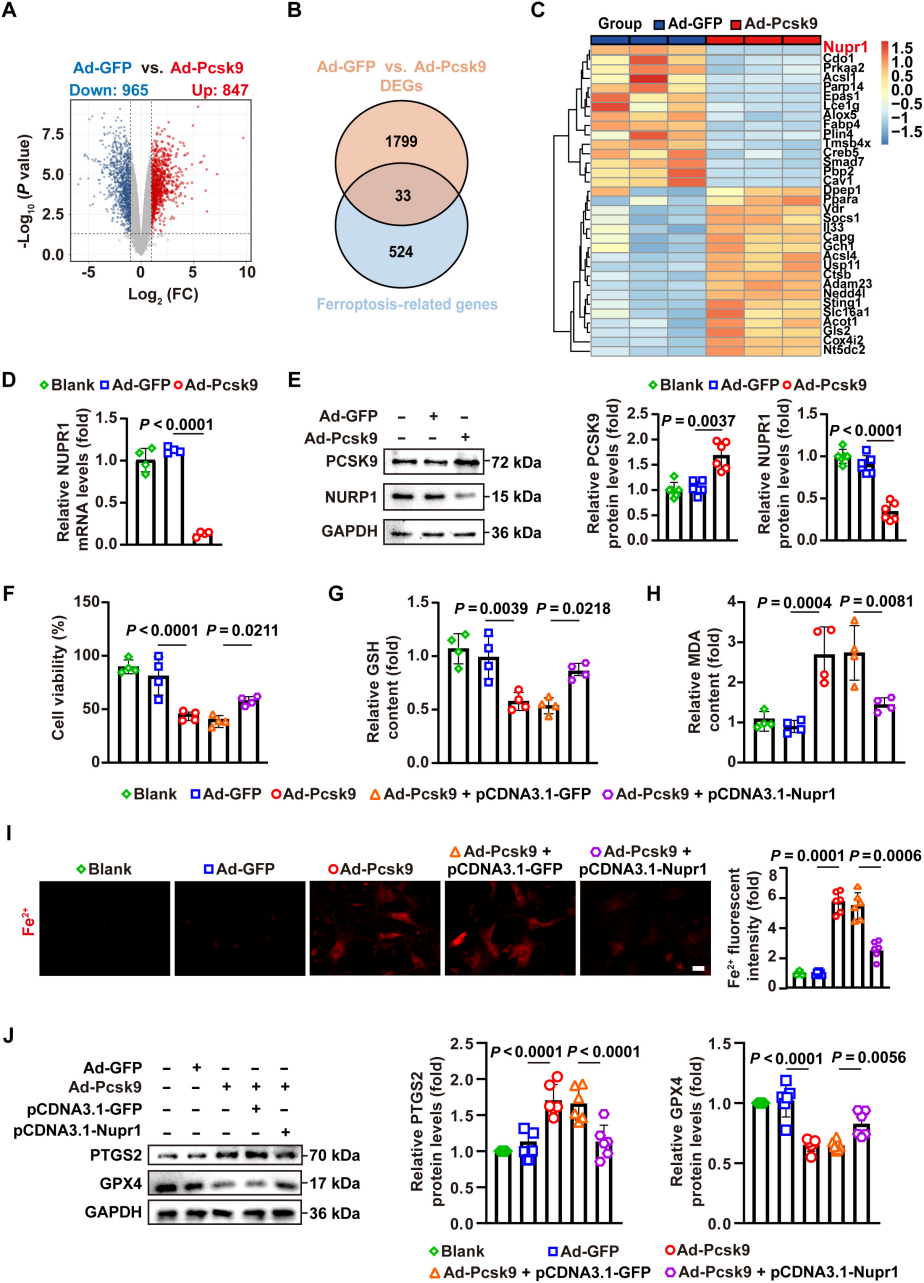
PCSK9 induces VSMC ferroptosis by reducing NUPR1 expression. (A) A volcano plot illustrating the differential gene expression analysis between the 2 groups (*n* = 3). (B) A Venn diagram displaying the overlap between differentially expressed genes (DEGs) and ferroptosis-related genes. (C) A heatmap representing the intersecting genes (*n* = 3). (D) Quantitative PCR analysis of NUPR1 mRNA levels in primary mouse VSMCs treated as indicated (*n* = 4). (E) Western blot analysis of the protein levels of PCSK9 and NUPR1 in primary mouse VSMCs treated as indicated (*n* = 6). (F to H) Measurement of cell viability (F), GSH levels (G), and MDA levels (H) in primary mouse VSMCs treated as indicated (*n* = 4). (I) Live cell FerroOrange staining in primary mouse VSMCs treated as indicated (*n* = 6). Scale bar: 50 μm. (J) Western blot analysis of the protein levels of PCSK9, PTGS2, and GPX4 in primary mouse VSMCs treated as indicated (*n* = 6). Statistical analysis was performed by 1-way ANOVA (D and E) or 2-way ANOVA test (F to J).

To explore the molecular mechanism by which PCSK9 regulates ferroptosis, we analyzed the RNA-seq data from PCSK9-overexpressing VSMCs. Out of 1,812 DEGs (847 up-regulated and 965 down-regulated; Fig. 4A), we found that NUPR1—a gene known to influence ferroptosis—was significantly down-regulated in PCSK9-overexpressing VSMCs (Fig. 4B and C). NUPR1 is a small, intrinsically disordered protein that is up-regulated under cellular stress and acts as a potent inhibitor of ferroptosis, playing a crucial role in maintaining redox homeostasis [27,28]. We confirmed that PCSK9 overexpression led to a marked reduction in NUPR1 at both the mRNA and protein levels in VSMCs (Fig. 4D and E). Furthermore, using the published single-cell dataset GSE155514, we identified 15 clusters by UMAP (Uniform Manifold Approximation and Projection) and annotated them into 9 major cell types—smooth muscle cells (SMCs), macrophages, fibrochondrocyte, endothelial cells (ECs), intermediate cell state, T cells, B cells, mast cells, and NK cells—using the Cardiovascular Cell Marker Database 2.0 (Fig. S4A). We then compared NUPR1 percentage expression and mean expression between stable and vulnerable plaque samples, finding that NUPR1 is down-regulated in SMCs from vulnerable carotid plaques (Fig. S4B). Consistently, immunofluorescence showed reduced NUPR1 in vulnerable versus stable human plaques (Fig. S4C).

To determine whether NUPR1 mediates the pro-ferroptotic effect of PCSK9, we performed rescue experiments by overexpressing NUPR1 in PCSK9-overexpressing VSMCs. Strikingly, restoring NUPR1 expression significantly reversed the ferroptosis-related effects of PCSK9 overexpression, as evidenced by normalized levels of GSH, MDA, Fe^2+^, GPX4, and PTGS2 (Fig. 4F to J). These findings suggest that PCSK9 induces ferroptosis in VSMCs at least in part by down-regulating the ferroptosis-suppressor NUPR1.

### PCSK9 enhances the lysosomal degradation of YAP1, leading to a reduction in NUPR1 expression in VSMCs

**Fig. 5.**
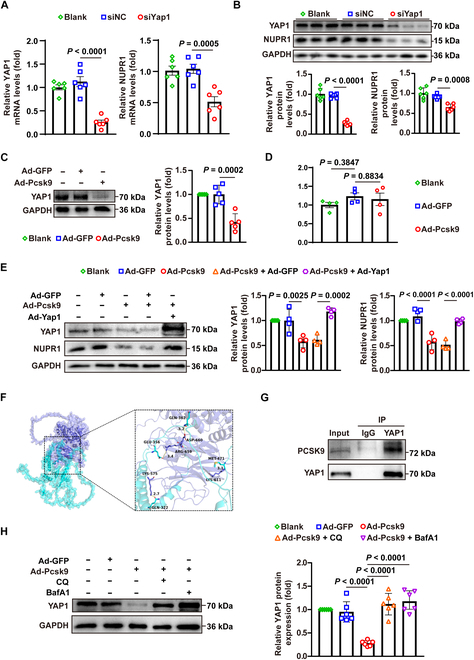
PCSK9 promotes lysosome-mediated degradation of YAP1, down-regulating NUPR1 expression in VSMCs. (A) qPCR analysis of YAP1 and NUPR1 mRNA levels in primary mouse VSMCs treated as indicated (*n* = 6). (B) Western blot analysis of YAP1 and NUPR1 protein levels in primary mouse VSMCs treated as indicated (*n* = 6). (C) Western blot analysis of YAP1 protein levels in primary mouse VSMCs treated as indicated (*n* = 5). (D) qPCR analysis of YAP1 mRNA levels in primary mouse VSMCs treated as indicated (*n* = 4). (E) Western blot analysis of YAP1 and NUPR1 protein levels in primary mouse VSMCs treated as indicated (*n* = 4). (F) Visualization of the PCSK9 protein as a dark blue model and the YAP1 protein as a cyan model in PyMOL, with their binding points represented as corresponding colored stick structures. (G) Co-immunoprecipitation of PCSK9 and YAP1 in primary mouse VSMCs. (H) Western blot analysis of YAP1 protein levels in primary mouse VSMCs treated with Ad-GFP or Ad-Pcsk9 and BafA1 (100 nM) for 6 h or treated with CQ (25 μM) for 6 h (*n* = 6). Statistical analysis was performed by 1-way ANOVA (A to D) or 2-way ANOVA test (E and H).

While it was clear that NUPR1 is down-regulated by PCSK9, the mechanism of this regulation was unclear. YAP1 knockdown reduced NUPR1 mRNA and protein in VSMCs (Fig. 5A and B), consistent with YAP1-dependent transcriptional regulation reported previously [29,30]. We also noticed that PCSK9 overexpression significantly reduced YAP1 protein levels in VSMCs, even though YAP1 mRNA levels were not markedly changed (Fig. 5C and D), hinting at post-translational regulation. To test whether PCSK9 regulates NUPR1 through YAP1, we established VSMCs co-overexpressing PCSK9 and YAP1. Notably, the suppression of NUPR1 caused by PCSK9 was largely reversed by YAP1 overexpression (Fig. 5E), indicating that PCSK9’s effect on NUPR1 is indeed YAP1-dependent.

We next investigated how PCSK9 reduces YAP1 protein. PCSK9 is known to bind certain intracellular proteins and target them for lysosomal degradation [31,32]. Using an in silico protein–protein docking analysis, we identified potential interfaces between PCSK9 and YAP1, and co-immunoprecipitation confirmed that PCSK9 physically interacts with YAP1 (Fig. 5F and G). We then treated PCSK9-overexpressing VSMCs with chloroquine or bafilomycin A1 (lysosomal inhibitors) to block lysosome-mediated degradation. Inhibition of lysosomal function led to a substantial restoration of YAP1 protein levels in the presence of PCSK9 overexpression (Fig. 5H), supporting the idea that PCSK9 promotes YAP1 degradation via the lysosomal pathway. Together, these data establish that PCSK9 interacts with YAP1 and accelerates its lysosomal turnover, thereby down-regulating YAP1 and consequently reducing NUPR1 expression.

### Degradation of PCSK9 via PROTAC suppresses ferroptosis in VSMCs

Given the detrimental effect of PCSK9 on VSMC survival and plaque stability, we explored a therapeutic strategy to neutralize PCSK9’s intracellular actions. Targeted protein degradation surpasses conventional pharmacological inhibition by catalytically eliminating the target, offering the potential for enhanced therapeutic outcomes and extended duration of action [33]. Building upon our established methodology[34], we employed an in silico rational design strategy to engineer the peptide-derived PCSK9 degrader, designated Cadd4. Cadd4 is a bifunctional molecule that links a PCSK9-binding peptide to the E3 ligase ligand ALAPYIP (with a fluorescent rhodamine tag) via a short linker, thereby recruiting the VHL E3 ubiquitin ligase to PCSK9 for its degradation (Fig. S5A).

**Fig. 6.**
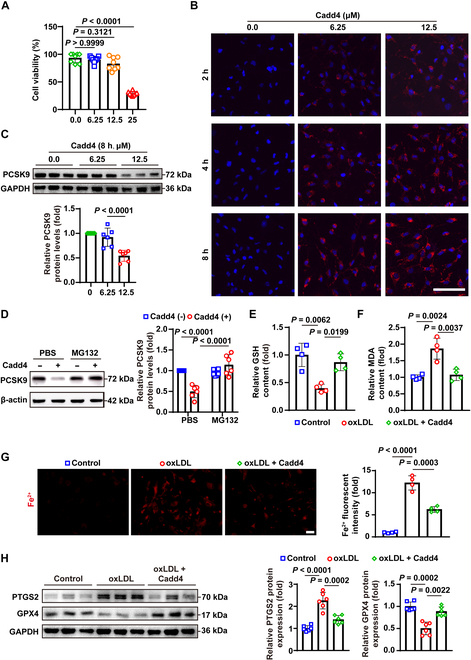
Cadd4 ubiquitinates and degrades PCSK9, inhibiting oxLDL-induced ferroptosis. Computer-aided drug design (CADD) techniques were applied to design PCSK9-targeted peptides, named Cadd4. (A) Cell viability in primary mouse VSMCs treated with Cadd4 at different concentration points (*n* = 8). (B) Fluorescent imaging showing the amount of Cadd4 entering VSMCs over time (red). (C) Western blot analysis of PCSK9 protein levels in primary mouse VSMCs treated with Cadd4 (8 μM) for 8 h (*n* = 6). (D) Western blot analysis of PCSK9 protein levels in primary mouse VSMCs treated with Cadd4 (8 μM) for 8 h and MG132 (5 μM) for 6 h (*n* = 6). (E and F) Measurement of GSH levels (E) and MDA levels (F) in primary mouse VSMCs treated with oxLDL (50 μg/ml) for 24 h and Cadd4 (8 μM) for 8 h (*n* = 4). (G) Live cell FerroOrange staining in primary mouse VSMCs treated with oxLDL (50 μg/ml) for 24 h and Cadd4 (8 μM) for 8 h (*n* = 4). Scale bar: 50 μm. (H) Western blot analysis of PTGS2 and GPX4 protein levels in primary mouse VSMCs treated with oxLDL (50 μg/ml) for 24 h and Cadd4 (8 μM) for 8 h (*n* = 6). Data are presented as mean ± SD. Statistical analysis was performed by Kruskal-Wallis test (A), 1-way ANOVA (C), or 2-way ANOVA test (D to G).

We first evaluated the biocompatibility and efficacy of Cadd4 in vitro. Cell viability assays showed that Cadd4 was well tolerated by VSMCs at low (6.25 μM) and medium (12.5 μM) concentrations, with some toxicity observed only at 25 μM (Fig. 6A). To assess target engagement, we treated VSMCs with 12.5 μM Cadd4 and found that it induced a marked reduction in PCSK9 protein levels within 8 h (Fig. 6B and C). This Cadd4-induced PCSK9 degradation was negated by co-treatment with the proteasome inhibitor MG132, confirming that Cadd4 acts through the ubiquitin–proteasome system (Fig. 6D). Functionally, Cadd4 treatment significantly elevated cellular GSH levels while reducing MDA accumulation and labile Fe^2+^ in oxidized LDL (oxLDL)-treated VSMCs (Fig. 6E to G). Consistently, Western blots showed that Cadd4 increased GPX4 and decreased PTGS2 expression in these cells (Fig. 6H). Together, these results indicate that inducing PCSK9 degradation with Cadd4 confers protection against oxLDL-induced ferroptosis in VSMCs.

### Degradation of PCSK9 via PROTAC maintains plaque stability

**Fig. 7.**
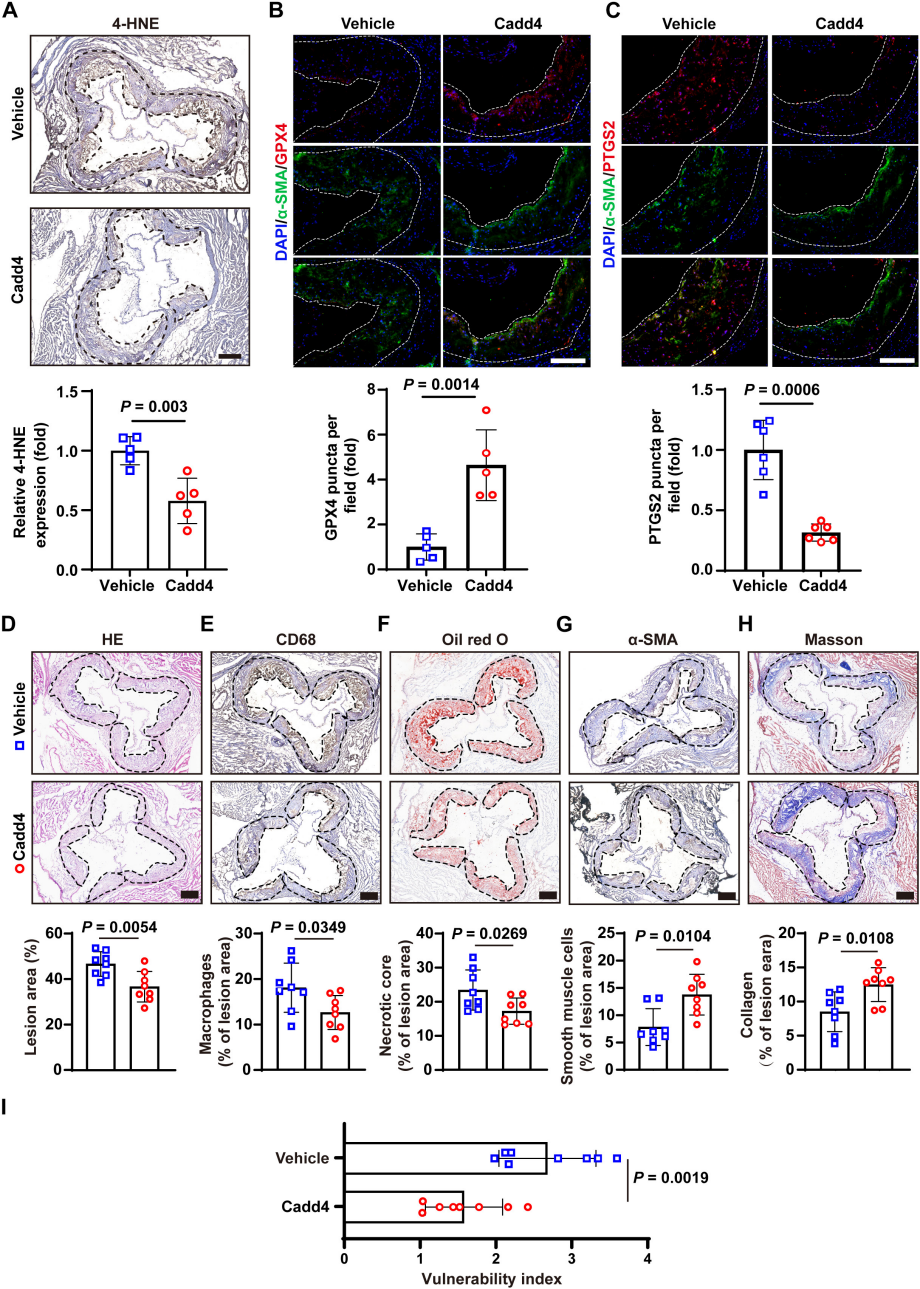
Degradation of PCSK9 via PROTAC restrains intraplaque VSMCs ferroptosis and increases plaque stability. (A) Immunohistochemical staining of 4-HNE (*n* = 5), unpaired *t* test. Scale bar: 200 μm. (B and C) Immunofluorescent staining of GPX4 and PTGS2 (green) alongside α-SMA (red) in mouse plaques, with nuclei stained by DAPI (blue) (*n* = 5 to 6). Scale bar: 150 μm. (D) Hematoxylin and eosin (H&E) staining of plaque lesion area expressed as a percentage of the vascular area (*n* = 8). Scale bar: 200 μm. (E) Immunohistochemical staining quantifying macrophages as percentages of the lesion area (*n* = 8). Scale bar: 200 μm. (F) Oil Red O staining of the necrotic core expressed as a percentage of the lesion area (*n* = 8). Scale bar: 200 μm. (G) Immunohistochemical staining quantifying smooth muscle cells as percentages of the lesion area (*n* = 8). Scale bar: 200 μm. (H) Masson staining for collagen content, expressed as a percentage of the lesion area (*n* = 8 mice). Scale bar: 200 μm. (I) Vulnerability index in aortic root plaques (*n* = 8). The solid continuous line denotes the area of the atherosclerotic plaque. Statistical analysis was performed by unpaired Student’s *t* test (A to I).

Encouraged by the in vitro results, we next evaluated the effects of Cadd4 in an atherosclerosis model. Seven-week-old Apoe^−/−^ mice were fed a Western diet and received intraperitoneal injections of Cadd4 (20 mg/kg, every other day) for 12 weeks (Fig. S5B). As expected, aortic root plaque analysis confirmed that Cadd4 effectively reduced PCSK9 protein levels in VSMCs in vivo (Fig. S5C). Interestingly, Cadd4 treatment did not significantly alter plasma triglyceride, HDL-C, or LDL-C levels, although total cholesterol was modestly reduced compared to controls (Fig. 7A to C). The finding of unaltered plasma lipids in Pcsk9-deficient Apoe^−/−^ mice is consistent with the notion that the lipid-modifying functions of PCSK9 exhibit differences between Apoe^−/−^ mice and human lipid metabolism [35]. More strikingly, Cadd4-treated mice exhibited biochemical evidence of ferroptosis suppression within their plaques: 4-HNE levels were significantly decreased, GPX4 expression was elevated, and PTGS2 expression was reduced in VSMCs of the aortic root lesions, relative to untreated mice (Fig. 7A to C).

We then examined whether Cadd4-mediated PCSK9 degradation translated into improved plaque stability. Morphometric analysis of aortic root plaques showed that Cadd4-treated mice had a significantly smaller total plaque area than vehicle-treated mice (Fig. 7D). Moreover, Cadd4 markedly decreased the CD68^+^ macrophage area and necrotic core size in plaques (Fig. 7E and F), while concomitantly increasing the α-SMA^+^ VSMC area and collagen content (Fig. 7G and H). Reflecting these changes, the plaque vulnerability index was significantly lower in the Cadd4 group (Fig. 7I). Collectively, these in vivo findings demonstrate that targeted degradation of PCSK9 via Cadd4 is an effective strategy for enhancing plaque stability. Consistent with our in vitro findings, Cadd4 increased YAP1 and NUPR1 expression in VSMCs of aortic root lesions compared with vehicle (Fig. S6A and B).

We then examined whether Cadd4-mediated PCSK9 degradation translated into improved plaque stability. Morphometric analysis of aortic root plaques showed that Cadd4-treated mice had a significantly smaller total plaque area than vehicle-treated mice (Fig. 7D). Moreover, Cadd4 markedly decreased the CD68^+^ macrophage area and necrotic core size in plaques (Fig. 7E and F), while concomitantly increasing the α-SMA^+^ VSMC area and collagen content (Fig. 7G and H). Reflecting these changes, the plaque vulnerability index was significantly lower in the Cadd4 group (Fig. 7I). Collectively, these in vivo findings demonstrate that targeted degradation of PCSK9 via Cadd4 is an effective strategy for enhancing plaque stability. Consistent with our in vitro findings, Cadd4 increased YAP1 and NUPR1 expression in VSMCs of aortic root lesions compared with vehicle (Fig. S6A and B).

## Discussion

Although we previously demonstrated that PCSK9-mediated inflammatory signaling in macrophages contributes to atherosclerosis development [36], in this study, we identified a novel role for VSMC-derived PCSK9 in promoting atherosclerotic plaque instability. We found that human plaques with higher PCSK9 expression exhibited clear hallmarks of vulnerability, and VSMC-specific PCSK9 overexpression in mice recapitulated these vulnerable plaque features without affecting systemic lipid levels, supporting the concept that PCSK9’s pro-atherogenic effects extend beyond its canonical role in LDL-C regulation. These findings are in line with emerging literature suggesting that PCSK9 contributes to cardiovascular pathology through lipid-independent mechanisms [36–38]. Indeed, PCSK9 has pleiotropic actions in other disease contexts—for example, its inhibition can enhance anti-tumor immune responses in cancer therapy—underscoring the diverse pathological roles of PCSK9 beyond cholesterol metabolism [39].

A key mechanistic insight of our work is the identification of ferroptosis, an iron-dependent form of regulated cell death, as a downstream consequence of PCSK9 activity in VSMCs. Ferroptosis is characterized by excessive lipid peroxidation, GSH depletion, and mitochondrial dysfunction, processes increasingly recognized as contributors to plaque instability [40–42]. Under both in vivo and in vitro conditions, elevating PCSK9 in VSMCs triggered multiple ferroptotic changes: accumulation of intracellular iron, increased levels of lipid peroxidation end products (4-HNE and MDA), heightened ROS, and dysregulated expression of ferroptosis-related enzymes (GPX4 suppression and PTGS2 up-regulation). Notably, knocking down PCSK9 or pharmacologically inhibiting ferroptosis mitigated these effects, indicating that PCSK9 is functionally a pro-ferroptotic factor in VSMCs. This mechanistic link advances our understanding of how PCSK9 exacerbates plaque damage at the cellular level. It is also noteworthy that the Hippo/YAP1 signaling pathway has been implicated in ferroptosis regulation in other contexts—for instance, hepatic deletion of HDAC3 activates Hippo signaling and disrupts iron homeostasis, leading to ferroptosis [43]—which aligns with our finding that YAP1 serves as a critical mediator of PCSK9-induced ferroptosis.

We further uncovered a novel PCSK9–YAP1–NUPR1 signaling axis that connects PCSK9 to ferroptotic cell death. Transcriptomic data pointed us to NUPR1, a stress-inducible protein that acts as an anti-ferroptotic factor [28,44], as being significantly down-regulated by PCSK9. Meanwhile, prior studies suggested that NUPR1 expression is positively regulated by YAP1 activity [26,45]. Our results confirmed and extended these findings: PCSK9 binds to YAP1 and accelerates its lysosomal degradation, thereby dampening YAP1’s transcriptional support of NUPR1. Overexpressing YAP1 rescued NUPR1 levels in the face of high PCSK9 and restoring NUPR1 itself protected VSMCs from ferroptosis despite PCSK9 overexpression. Thus, we establish that PCSK9 promotes ferroptosis and led to plaque instability through a previously unrecognized YAP1–NUPR1 pathway.

Building on these insights, we evaluated a therapeutic approach to counteract PCSK9’s deleterious effects. Conventional PCSK9 inhibitors (monoclonal antibodies) act extracellularly to block PCSK9’s interaction with LDLR, but they do not address PCSK9’s intracellular actions. Here, we show that a cell-permeable PROTAC-based PCSK9 degrader (Cadd4) can effectively eliminate intracellular PCSK9, thereby blunting VSMC ferroptosis and slowing plaque progression in an atherosclerotic mouse model. Compared to other reported targeted therapies for atherosclerosis, such as nanoparticle-based systems designed to deliver anti-inflammatory or antioxidant agents to plaques [46,47], PROTACs offer a compelling advantage by harnessing the cell’s protein degradation machinery to remove the target protein [33], and in the case of Cadd4, this translated into increased fibrous cap thickness and reduced necrotic core and inflammation in plaques. Cadd4, therefore, represents a proof-of-concept therapeutic that addresses PCSK9’s role in plaque destabilization from inside the cell, beyond its effect on plasma lipids. Moreover, the potential synergy between nanovector-driven targeted delivery and intracellular degraders like Cadd4 should not be overlooked. Future combination regimens could leverage the precise tissue tropism of nanomaterials to deliver degraders more efficiently, enabling tailored therapy based on specific plaque phenotypes.

Despite the clear evidence supporting PCSK9’s pro-ferroptotic, plaque-destabilizing actions, our study has some limitations. First, while Cadd4 showed efficacy in mice, its pharmacokinetic profile, potential off-target effects, and safety in larger animals or humans remain to be investigated before clinical translation. Besides, our study did not investigate the effects of Cadd4 and PCSK9 inhibitors. While PCSK9 monoclonal antibodies target extracellular PCSK9, Cadd4 can eliminate intracellular PCSK9. The therapeutic and potential synergistic effects of these 2 agents on atherosclerosis warrant further evaluation. Second, our previous work suggested that YAP1 can inhibit VSMC ferroptosis by maintaining glutaminase (GLS1) and GSH synthesis [26], but we did not explore the YAP1–GLS1 interaction in depth here. These aspects will be important to address in subsequent studies. Third, the sample size of human atherosclerotic specimens was relatively small, and future studies with larger cohorts are needed to validate our findings.

In conclusion, our study uncovers a novel mechanism by which PCSK9 promotes atherosclerotic plaque instability through the induction of ferroptosis in VSMCs. We demonstrate that PCSK9 exacerbates plaque vulnerability independent of systemic lipid levels, acting through a newly identified YAP1–NUPR1 axis: PCSK9-mediated degradation of YAP1 suppresses NUPR1, thereby heightening VSMC susceptibility to ferroptosis. Furthermore, we provide proof of principle that targeted degradation of PCSK9 (using the Cadd4 PROTAC) can effectively stabilize plaques by reversing these pathological processes. These findings open new avenues for treating vulnerable atherosclerotic plaques by targeting the nontraditional, intracellular actions of PCSK9.

## Materials and Methods

### Human atherosclerotic plaque samples

The use of human tissue was approved by the Medical Ethics Committee of the University of South China, and written informed consent was obtained from all donors, in accordance with the Declaration of Helsinki. Autopsy specimens of human aortic plaques were obtained from the University of South China Forensic Identification Center, spanning a range of atherosclerotic severity (see Table S1 for clinical details).

### Mouse models

All animal experiments were approved by the Institutional Animal Care and Use Committee of the University of South China (Approval No. USC2023XS099). VSMC-specific PCSK9 overexpression mice were generated on a C57BL/6 background using a Cre-loxP system. Briefly, a Rosa26-loxP-stop-loxP-mPCSK9 construct was engineered and used to create PCSK9^flox/flox^ mice. These mice were then crossed with SM22α-Cre transgenic mice. Offspring positive for SM22α-Cre (which deletes the loxP-flanked stop codon) were used as the PCSK9^SMC OE^ group, while Cre-negative littermates (carrying PCSK9^flox/flox^ but no Cre) served as controls.

To model atherosclerosis more faithfully, we employed an established approach by injecting rAAV8-D377Y-mPCSK9 (5 × 10^11^ viral genomes in 200 μl of saline) [19,48,49], followed by a 12-week HFD (see Fig. S2A).

For therapeutic experiments, 7-week-old male Apoe^−/−^ mice were fed an HFD for 12 weeks and concurrently treated with the PCSK9-targeted PROTAC Cadd4 or vehicle. The Cadd4 group (*n* = 8) received 20 mg/kg Cadd4 via intraperitoneal injection every 2 days, while the control group (*n* = 8) received equivalent injections of 0.9% saline. Both groups remained on HFD throughout the 12-week treatment period. Mice were anesthetized with 1% isoflurane for blood collection and were humanely euthanized with 4% isoflurane prior to tissue harvest. All procedures followed institutional guidelines and conformed to the NIH Guide for the Care and Use of Laboratory Animals.

### Cell culture and treatments

Primary mouse aortic VSMCs were isolated and cultured in Dulbecco’s Modified Eagle Medium with 10% fetal bovine serum at 37 °C in a humidified 5% CO_2_ incubator. For all experiments, cells were serum-starved for 4 to 6 h before treatment. VSMCs were then treated with 50 μg/ml of oxLDL (Yiyuan Biotech) for 24 h to induce lipid stress. Where indicated, bafilomycin A1 (100 nM, 6 h pre-treatment) or chloroquine (25 μM, 6 h) was used to inhibit autophagic–lysosomal degradation. To block proteasomal degradation, cells were treated with the proteasome inhibitor MG132 (10 μM) during the experimental period. Adenoviral vectors for PCSK9 or control (empty vector) were applied at a multiplicity of infection of 50 for 48 h to achieve overexpression. For NUPR1 rescue experiments, a plasmid encoding mouse NUPR1 was transiently transfected into VSMCs using Lipofectamine 3000, 24 h prior to analyses.

### Histological analysis

Aortic root specimens from mice were fixed in 4% paraformaldehyde, embedded in Optimal Cutting Temperature Compound or paraffin, and sectioned (4 to 6 μm thickness). Sections for Masson’s trichrome staining were processed using a commercial kit (Solarbio G1346) following the manufacturer’s protocol. Briefly, sections were stained with hematoxylin for 8 min, rinsed in water, and differentiated in 0.5% to 1% hydrochloric acid. After thorough washing, sections were stained in Masson’s solution for 10 min, then treated with 1% phosphotungstic acid for 10 min. Next, sections were counterstained with aniline blue for 5 min, followed by a brief rinse in 1% acetic acid (1 min). Finally, sections were dehydrated in graded ethanol (95% and absolute), cleared in xylene, and mounted in neutral balsam. For H&E staining, a standard kit (Solarbio G1120) was used. Sections were rehydrated in distilled water for 10 min, stained in hematoxylin for 1 min, and “blued” under running tap water for ~2 min. After a quick dip in eosin (1 to 3 s), sections were rinsed in water, dehydrated, and transparent.

### IHC and immunofluorescence

Paraffin sections were deparaffinized and subjected to heat-mediated antigen retrieval (sodium citrate buffer) using a microwave (high power, 3 min). To mitigate endogenous peroxidase activity (specifically for IHC), sections were treated with 3% H₂O₂, followed by blocking in 10% bovine serum albumin to minimize nonspecific binding. For IHC, we used a 2-step IHC detection kit (Maixin Biotech KIT-9710) according to the manufacturer’s instructions. Sections were incubated with primary antibodies (anti-PCSK9, anti-α-SMA, anti-CD68, etc.) at 4 °C overnight. The next day, horseradish peroxidase-conjugated secondary antibodies from the kit were applied, and signal was developed with a diaminobenzidine chromogen solution. Nuclei were counterstained with hematoxylin, and slides were mounted for microscopic evaluation. For immunofluorescence staining, sections or cultured cells were incubated with primary antibodies (e.g., PCSK9, GPX4, and PTGS2) overnight, followed by appropriate secondary antibodies, and nuclei were stained with 4′,6-diamidino-2-phenylindole (DAPI). Stained sections were observed under a fluorescence microscope, and images were captured for analysis. (Additional details on antibodies, reagents, and quantitative analysis methods are provided in the Supplementary Materials.)

## Data Availability

The underlying data are available in the article and the Supplementary Materials.
