# Supplementary figures and images for: PCSK9 Promotes Atherosclerotic Plaque Instability by Inducing VSMC Ferroptosis through the YAP1–NUPR1 Axis
Source: Research (Wash D C). 2025 Oct 7;8:0922. doi: 10.34133/research.0922 (PMC12501616; doi:10.34133/research.0922)

## A

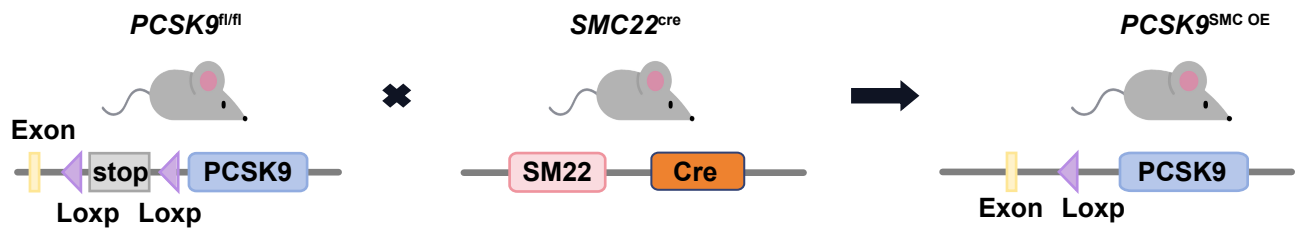

## B

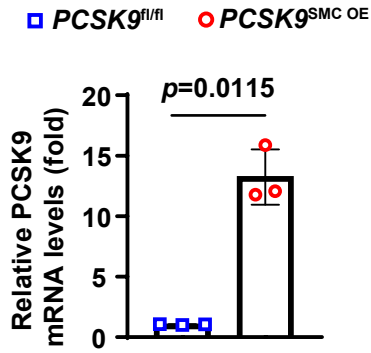

## C

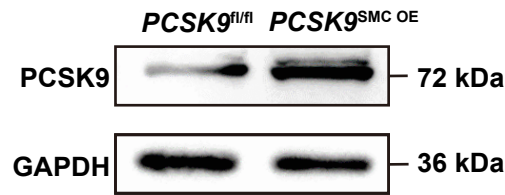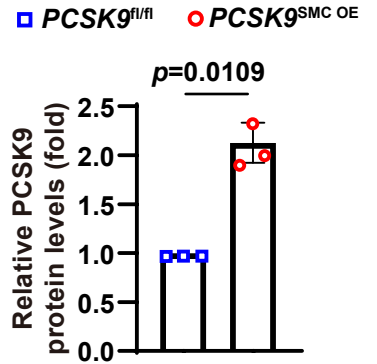

## D

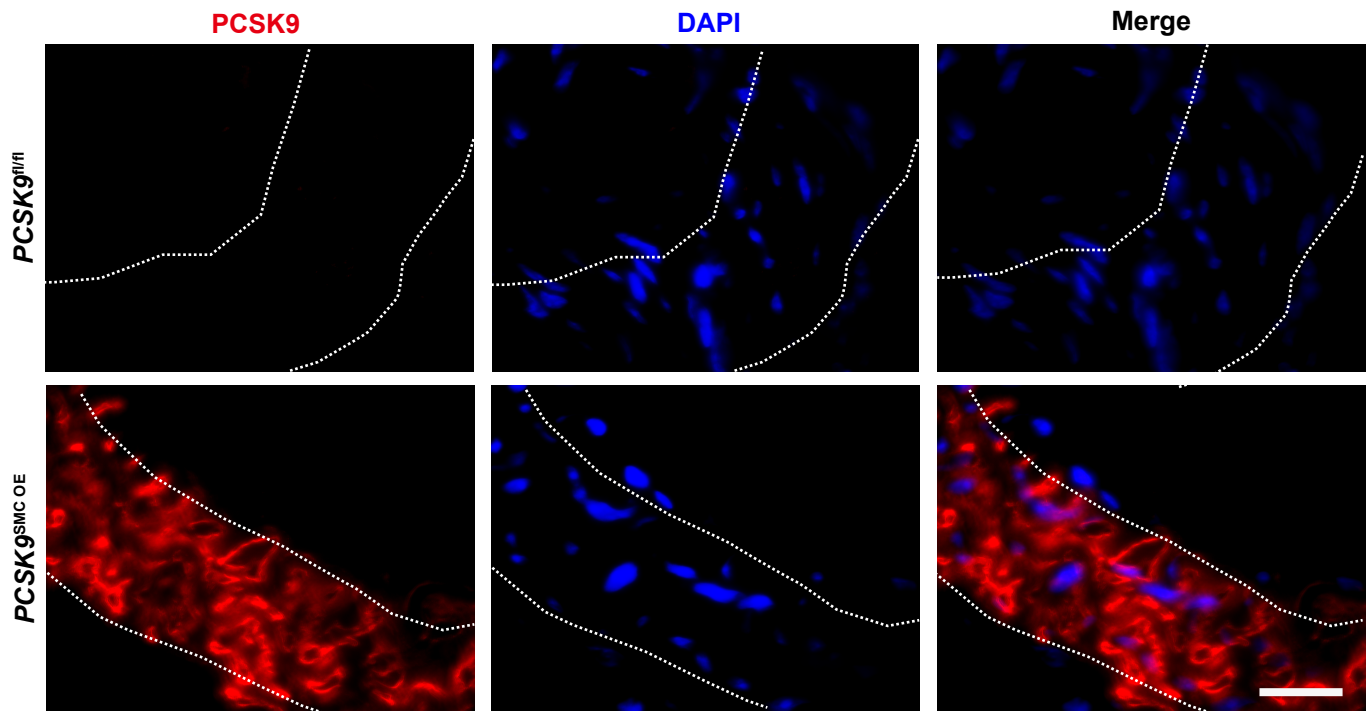

Supplement: Supplementary 1 — Figs. S1 to S6 [file research.0922.f1.zip › Supplementary figure 1.pdf]

## A

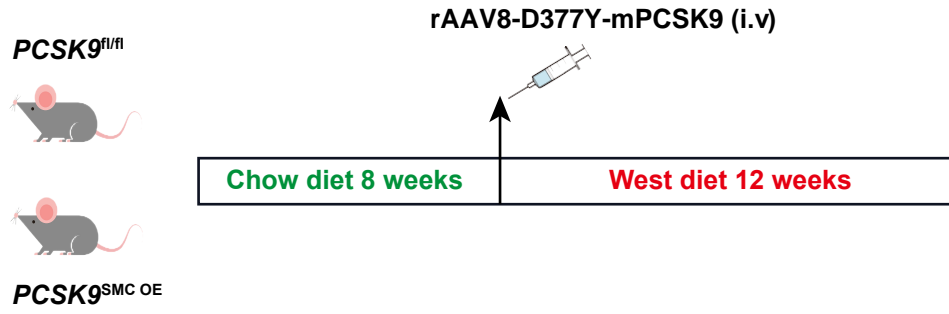

## B

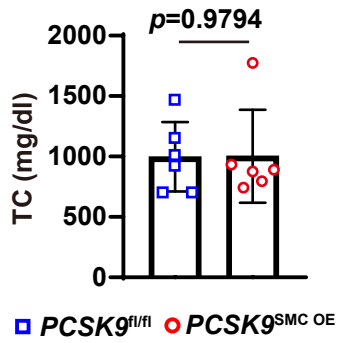

## C

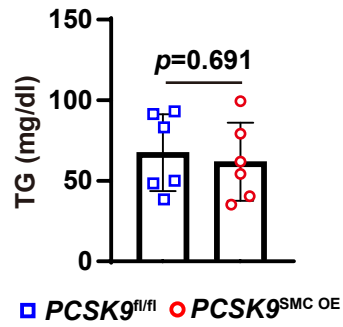

## D

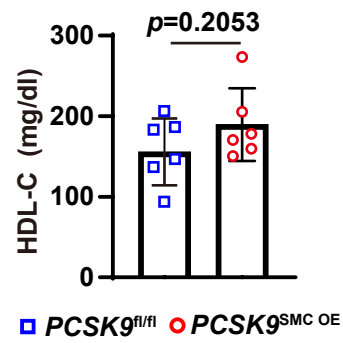

## E

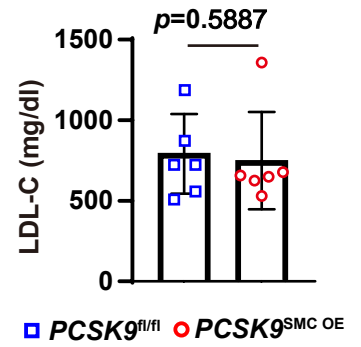

Supplement: Supplementary 1 — Figs. S1 to S6 [file research.0922.f1.zip › Supplementary figure 2-new(1).pdf]

A

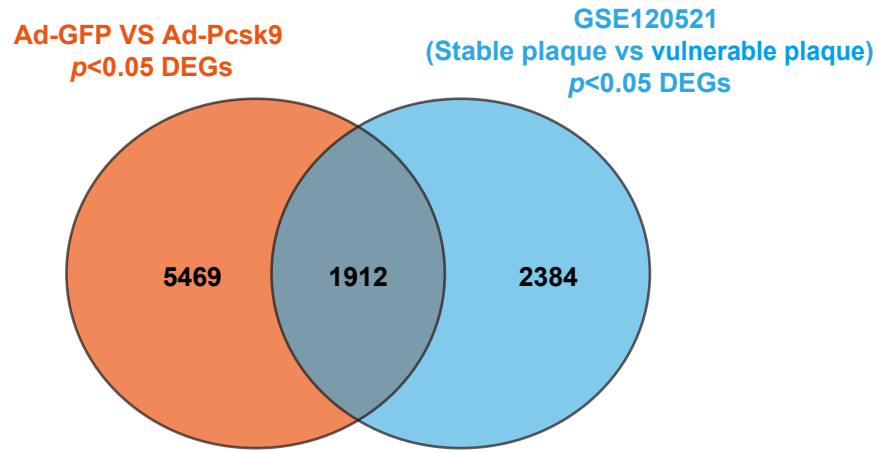

B

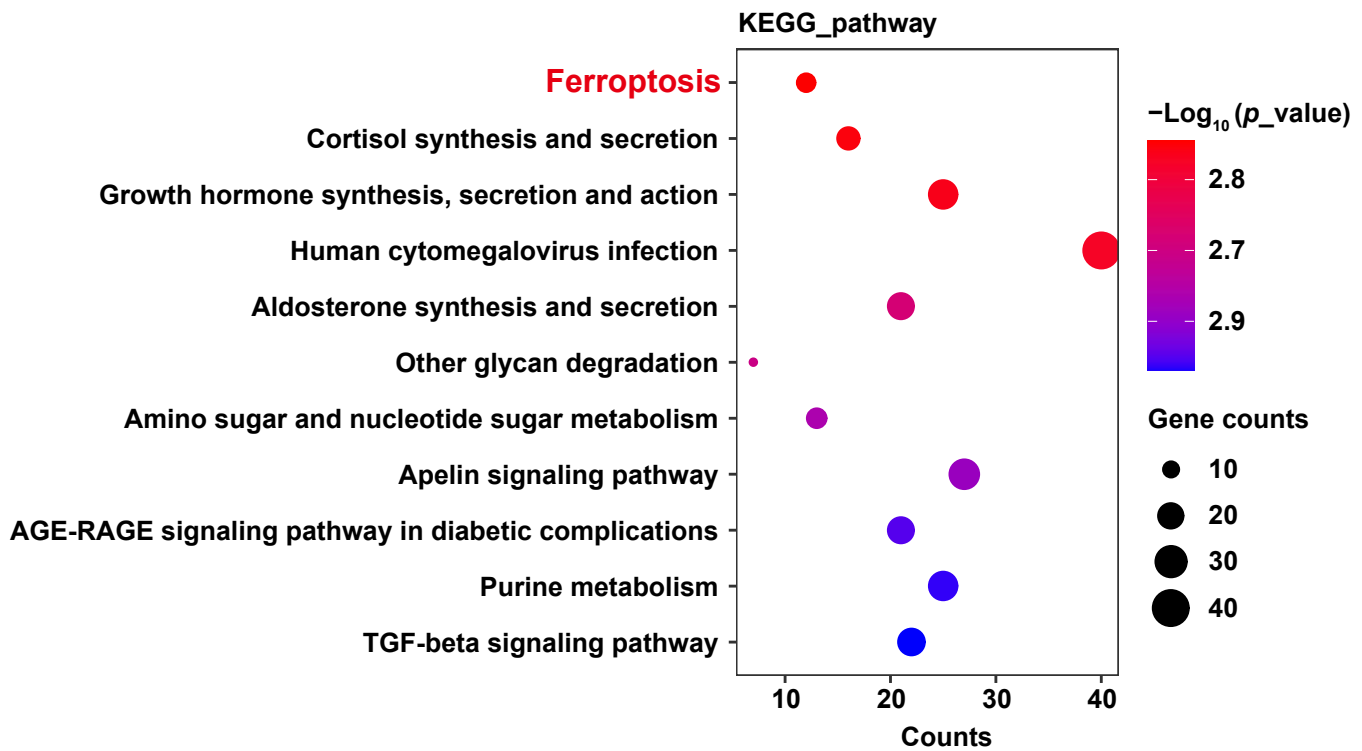

Supplement: Supplementary 1 — Figs. S1 to S6 [file research.0922.f1.zip › Supplementary figure 3.pdf]

**A**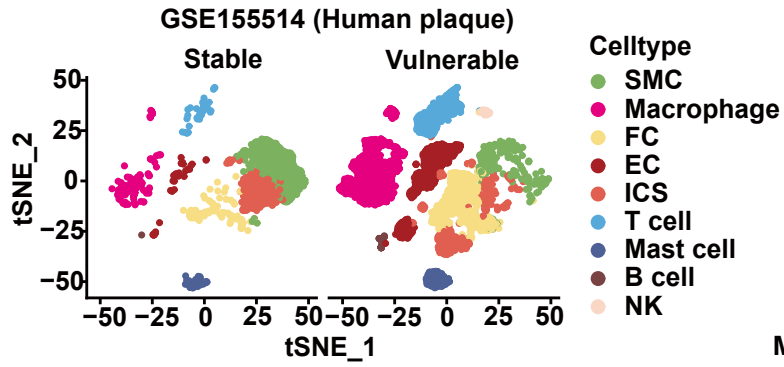**B**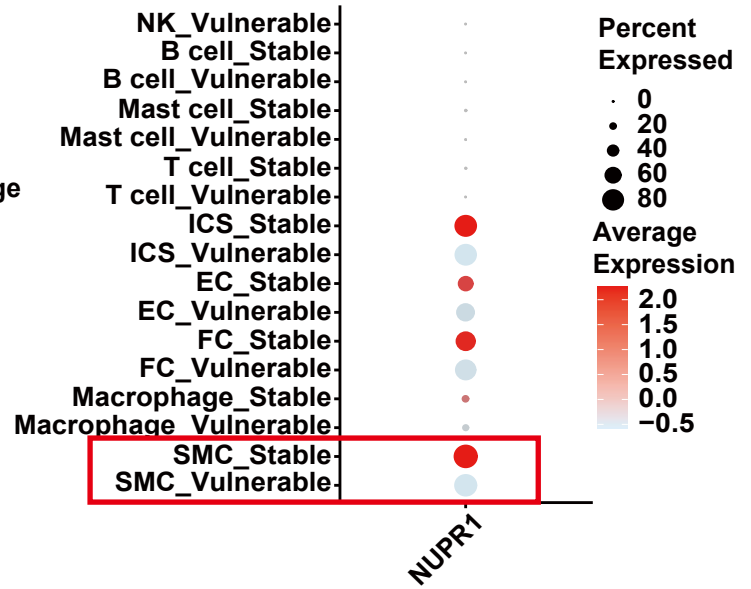**C**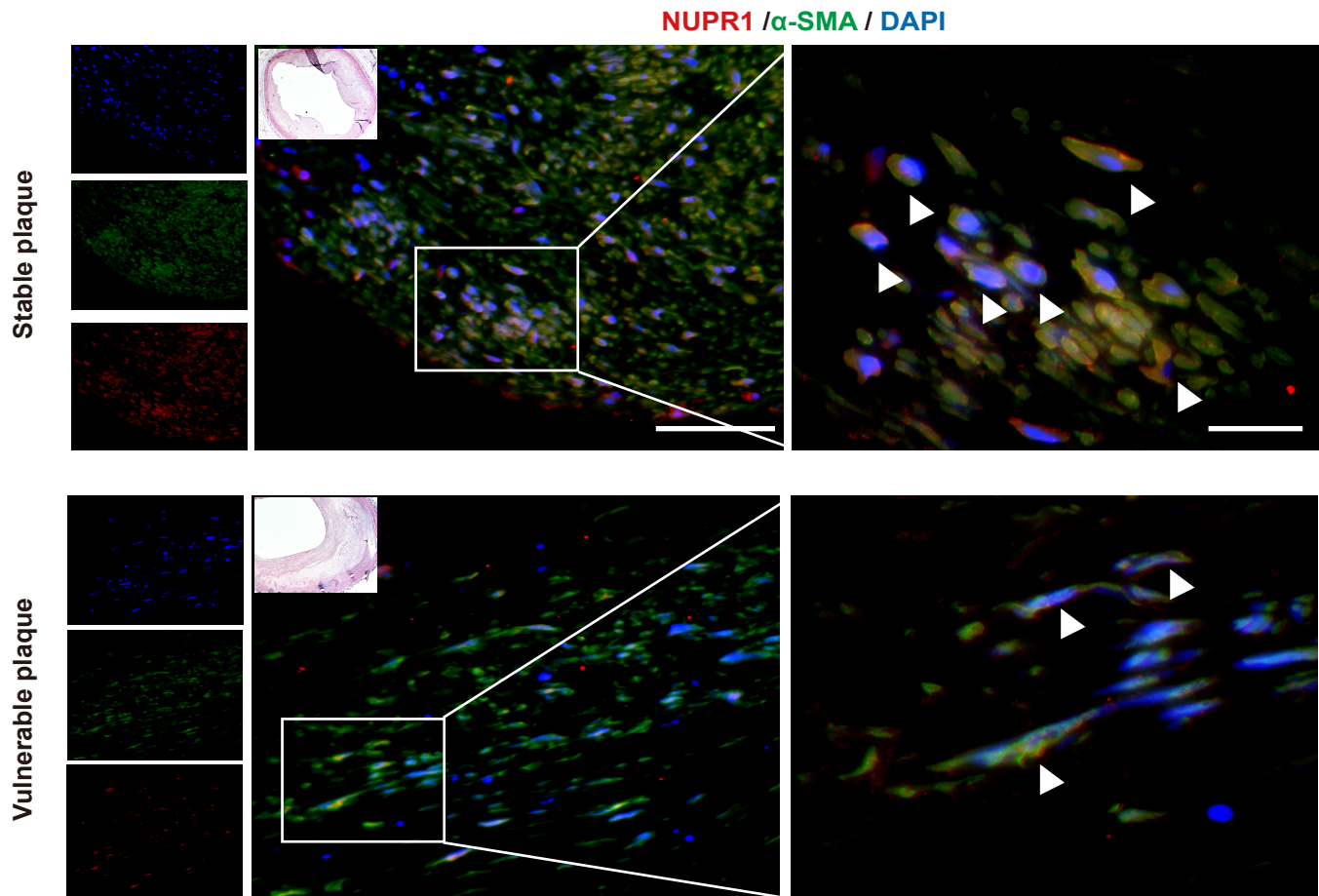

Supplement: Supplementary 1 — Figs. S1 to S6 [file research.0922.f1.zip › Supplementary figure 4-new.pdf]

**A**

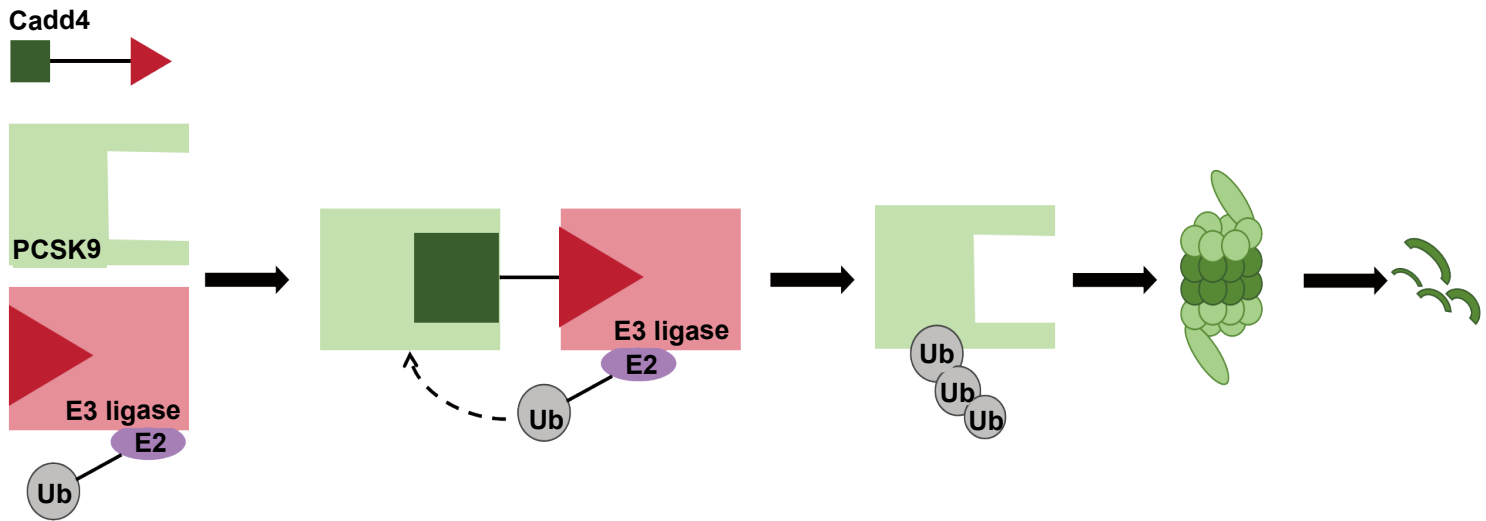

**B**

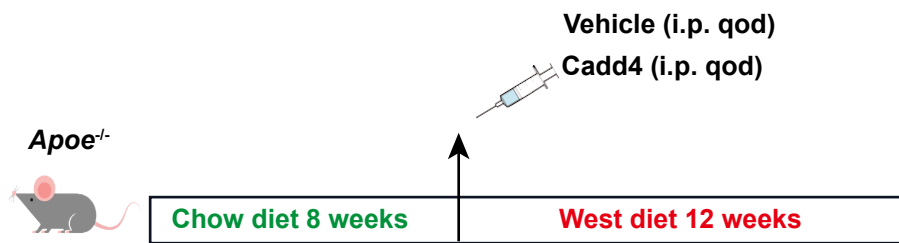

**C**

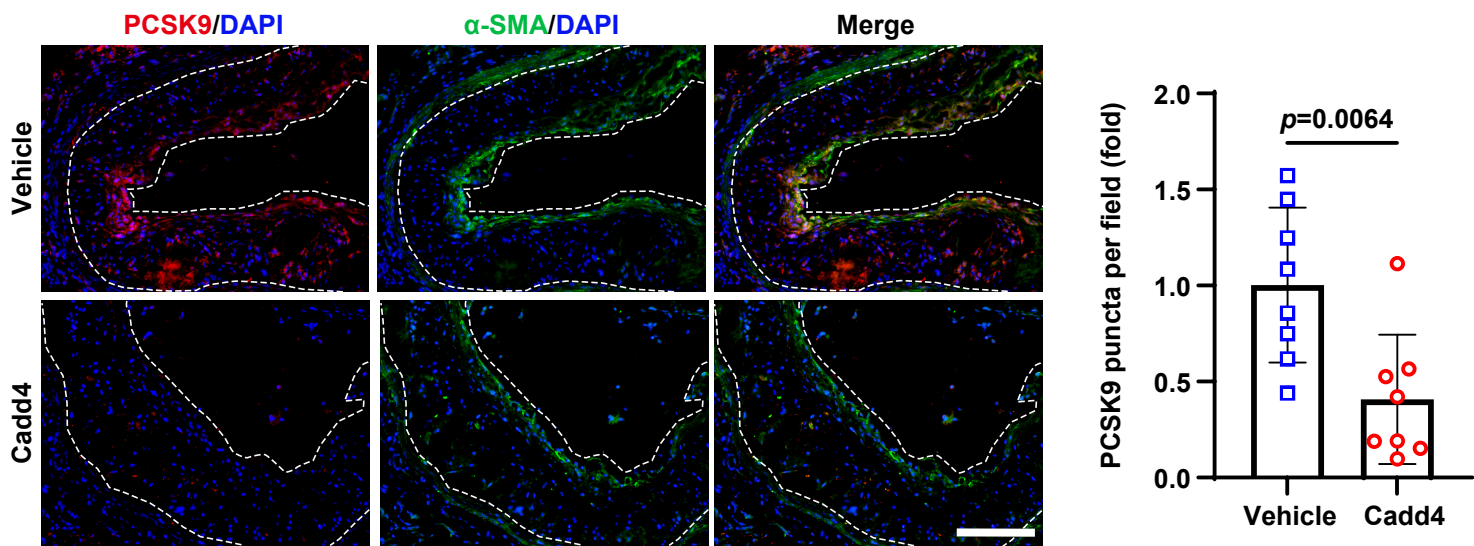

**D**

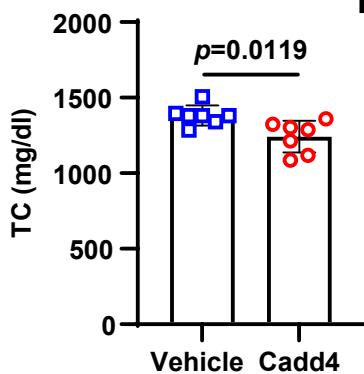

**E**

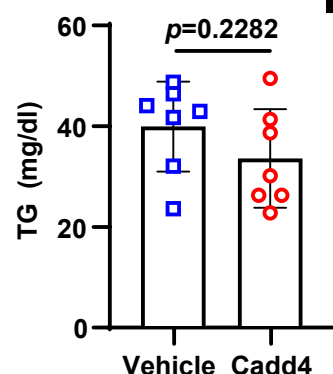

**F**

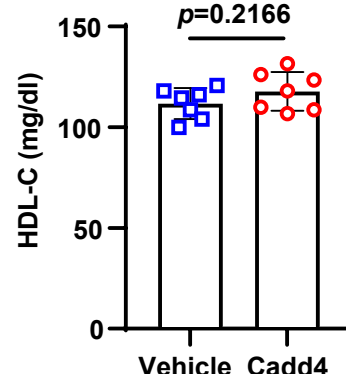

**G**

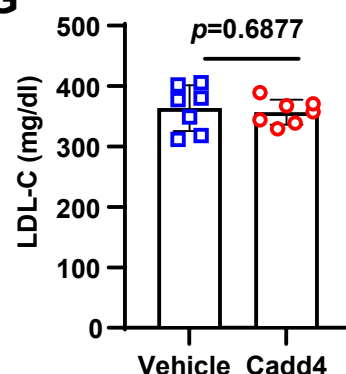

Supplement: Supplementary 1 — Figs. S1 to S6 [file research.0922.f1.zip › Supplementary figure 5.pdf]

**A**

YAP1

$\alpha$ -SMA

DAPI

Merge

Vehicle

Cadd4

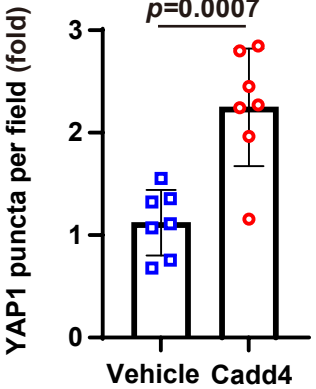

**B**

NUPR1

$\alpha$ -SMA

DAPI

Merge

Vehicle

Cadd4

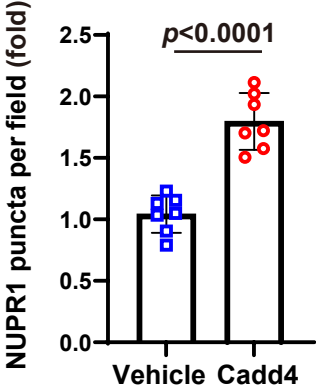

Supplement: Supplementary 1 — Figs. S1 to S6 [file research.0922.f1.zip › Supplementary figure 6-new.pdf]
